# Metabolic phenotypes and risk of colorectal cancer: a systematic review and meta-analysis of cohort studies

**DOI:** 10.1186/s12885-021-09149-w

**Published:** 2022-01-21

**Authors:** Golnoosh Goodarzi, Hadis Mozaffari, Tahereh Raeisi, Fatemeh Mehravar, Bahman Razi, Maryam Lafzi Ghazi, Nazila Garousi, Shahab Alizadeh, Parisa Janmohammadi

**Affiliations:** 1 Department of Nutrition, Science and Research Branch, Islamic Azad University, Tehran, Iran; 2 Faculty of Land and Food Systems, University of British Columbia, Vancouver, Canada; 3 Department of Medicine, Hormozgan University of Medical Sciences, Bandar Abbas, Iran; 4 Department of Epidemiology and Biostatistics, School of Public Health, Tehran University of Medical Sciences (TUMS), Tehran, Iran; 5 Department of Hematology, Faculty of Medical Sciences, Tarbiat Modares University, Tehran, Iran; 6 Department of Exercise Physiology, Central Tehran Branch, Islamic Azad University, Tehran, Iran; 7 Department of Clinical Nutrition, School of Nutrition and Food Science, Isfahan University of Medical Sciences, Isfahan, Iran; 8 Department of Clinical Nutrition, School of Nutritional Sciences and Dietetics, Tehran University of Medical Sciences (TUMS), Tehran, Tehran Province Iran

**Keywords:** Obesity, Colorectal cancer, Phenotypes

## Abstract

**Background:**

The association of obesity with colorectal cancer (CRC) may vary depending on metabolic status.

**Objective:**

This meta-analysis aimed to investigate the combined impacts of obesity and metabolic status on CRC risk.

**Methods:**

The Scopus, PubMed, and web of sciences databases were systematically searched up to Jun 2021 to find all eligible publications examining CRC risk in individuals with metabolically unhealthy normal-weight (MUHNW), metabolically healthy obesity (MHO), and metabolically unhealthy obesity (MUHO) phenotypes.

**Results:**

A total of 7 cohort studies with a total of 759,066 participants were included in this meta-analysis. Compared with healthy normal-weight people, MUHNW, MHO, and MUHO individuals indicated an increased risk for CRC with a pooled odds ratio of 1.19 (95% CI = 1.09–1.31) in MUHNW, 1.14 (95% CI = 1.06–1.22) in MHO, and 1.24 (95% CI = 1.19–1.29) in MUHO subjects. When analyses were stratified based on gender, associations remained significant for males. However, the elevated risk of CRC associated with MHO and MUHO was not significant in female participants.

**Conclusions:**

The individuals with metabolic abnormality, although at a normal weight, have an increased risk for CRC. Moreover, obesity is associated with CRC irrespective of metabolic status.

**Supplementary Information:**

The online version contains supplementary material available at 10.1186/s12885-021-09149-w.

## Introduction

Colorectal cancer (CRC) is the third most common cancer in men and the second one in women; over 1.8 million new cases of CRC were recorded in 2018 [1]. In recent years, the increased incidence of CRC to a high extent is related to epidemiological and nutritional changes as well as the Western lifestyle [2]. A meta-analysis study indicated that the Western dietary pattern increases the risk of CRC. Obesity, which is closely associated with the Western dietary pattern, is also a risk factor for colorectal cancer [3, 4]. The majority of patients with obesity share common metabolic abnormalities, namely hyperglycemia, insulin resistance, abdominal obesity, hyperlipidemia, and hypertension. Metabolic abnormalities have been postulated to explain the role of obesity in the development of CRC [5]. A growing number of evidence from epidemiological studies shows that not all individuals with obesity have metabolic abnormalities, a phenomenon known as metabolically healthy obesity (MHO). Likewise, not all individuals with normal weight are metabolically healthy, a phenomenon known as metabolically unhealthy normal weight (MUNW) [6].. Accordingly, this concept has been recently taken into consideration and different body size phenotypes have been defined based on the metabolic health status [7]. Metabolic phenotypes are the consequence of the interactions between different factors including dietary, lifestyle, environmental factors, genetic factors, and microbial factors [8]. Individuals are classified into the following different metabolic phenotypes including the metabolically healthy normal weight (MHNW), metabolically unhealthy normal weight (MUHNW), metabolically healthy obese (MHO), the prevalence of this phenotype, according to the definitions of obesity and metabolic health, varies from 6 to 38.4% among different populations [9] These people express a favorable metabolic profile, are insulin sensitive, and express an optimal lipid profile, fat distribution and low levels of systemic inflammatory responses [10]. Another phenotypes are metabolically unhealthy obese (MUHO) [11] and metabolically unhealthy and normal-weight (MUNW). The BMI of MUNW individuals is less than 25, but they express metabolic abnormalities such as increased levels of adiposity, insulin resistance, higher susceptibility to type 2 diabetes, and cardiovascular diseases [12]. Kabat et al. (2018) indicated that, compared to metabolically healthy individuals with normal weight, the MUNW phenotype increases the risk of CRC in postmenopausal women [13]. The simultaneous effect of obesity and MetS on CRC has been discussed in previous observational studies and the results were controversial so far [14–16]. A recent meta-analysis showed that participants with MHO had a higher risk of cancer (of any type) than those with metabolically healthy normal weight (MHNW) or metabolically healthy non-obesity (included overweight, normal weight, and underweight) [17]. However, this meta-analysis combined different types of cancer in a single analysis. The influence of metabolic obesity phenotypes on the risk of cancer may differ according to the cancer site. Therefore, it may not be appropriate to analyze cancer at different sites as a single exposure. Moreover, MUNW and metabolically unhealthy obesity (MUHO) was not investigated in the prior meta-analysis. Given the considerations mentioned above, we performed a meta-analysis of prospective observational studies to clarify whether MHO, MUHO, or MUNW (compared with MHNW) is associated with CRC risk.

## Methods

In the present meta-analysis, the Preferred Reporting Items for Systematic Reviews and Meta-Analyses 2020 (PRISMA) statement was followed to write and report data [18]. This study does not contain any studies with human participants or animals carried out by any of the authors.

### Search strategy

A systematic literature search was conducted through three major databases including PubMed, Scopus, and Web of Science up to Jun 2021. The systematic search was supplemented by a screening of the reference lists of all eligible studies and reviews. The combination of the following controlled vocabulary term was searched: (Obesity [Mesh] OR “Body Mass Index” [Mesh] OR BMI OR obese OR overweight OR “normal weight” OR non-obese OR non-obese) AND (metabolic OR metabolically OR healthy OR unhealthy OR benign OR Abnormal) AND (Colorectal OR colon OR Rectal OR rectum) AND (Neoplasms [Mesh] OR Neoplasia OR Neoplasm* OR cancer OR carcinoma OR Colorectal Neoplasms OR Colonic Neoplasms OR Rectal Neoplasms OR tumor*). The primary search was not restricted to the language, ethnicity, or geographical region.

### Inclusion and exclusion criteria

All relevant studies considered the following criteria were included: 1) studies with prospective design (prospective cohort, nested case-control and case-cohort); 2) examined association of metabolically healthy obese (MHO), metabolically unhealthy normal weight (MUHNW) and metabolically unhealthy obese (MUHO) phenotypes of body size with the risk of CRC; 3) stratified participants according to metabolic status and BMI categories and had one reference group in the MHNW category; 4) reported the definition of being metabolically healthy; 5) studies which reported risk estimates (relative risk (RR) or hazard ratio (HR) or odds ratio (OR)) and the corresponding 95% confidence intervals (CIs) or sufficient data to calculate them. Letters, comments, reviews, short communication, case reports, book chapters, and studies conducted on animals all were excluded.

### Data extraction and quality assessment

The required data were extracted independently by two authors according to a standardized form for the following information: the first author’s name, year of publication, country of origin, sex, mean or range of age, sample size, and risk estimates with their 95% CIs, confounding factors adjusted for in the analyzes, and criteria used to define metabolically healthy status. If there was any discrepancy between the two authors the extracted data were compared with the original file. A quality assessment of included studies was carried out by two reviewers. To evaluate the methodological quality of eligible studies, Newcastle-Ottawa Scale (NOS) [19] was applied. The NOS is a star system scoring studies based on selection, comparability, and outcome parameters.

### Statistical analysis

In this meta-analysis, estimated pooled ORs with 95% CIs were used to assess the strength of association between metabolic phenotypes of obesity and risk of CRC and the metabolically healthy normal weight (MHNW) was considered as the reference group. Statistical heterogeneity was assessed using the Chi-square test (*p* < 0.1) and calculation of the I2 statistic. Accordingly, heterogeneity was significant if Q statistic had p < 0.1 or if I^2^ > 50%. Low heterogeneity (≤ 25%), moderate heterogeneity (> 25 to 50%), and high heterogeneity (> 50%) were also evaluated. Data were combined via the random-effects model (REM) and fixed-effect models when appropriate. To find and attenuate potential sources of heterogeneity subgroup analysis by gender (male/female) was performed. The conclusiveness and robustness of results by excluding each of the studies from the pooled estimate and analyzing the rest of them were evaluated. The publication bias was evaluated through visual inspection of asymmetry, and Egger’s weighted regression test (*p*-value less than 0.05 considered significant). Study characteristics and data were extracted to RevMan 5 (Review Manager, version 5.3; The Cochrane Collaboration, 2015) and STATA version 17.0 (Stata Corporation, College Station, TX).

## Results

### Findings from the systematic review

**Fig. 1:**
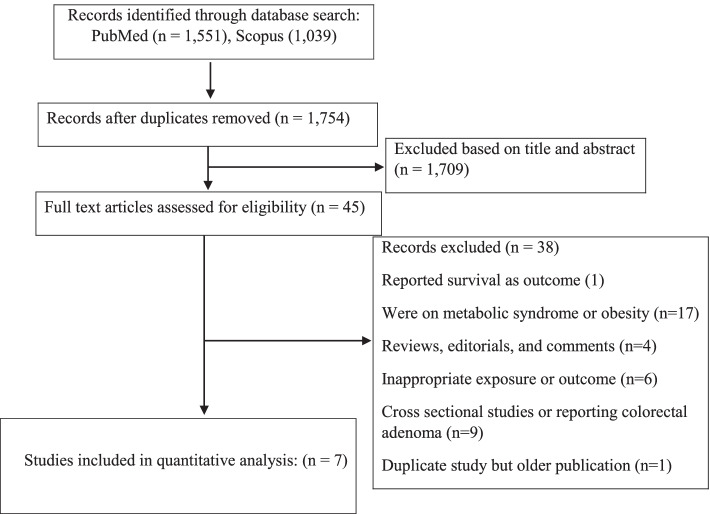
Flow diagram of the study

A total of 2590 publications were obtained by the systematic literature search. The flow chart indicating the process of screening of studies is reported in Fig. 1. Finally, 7 cohort studies [13, 20–25], with a total sample size of 759,066 participants met eligibility criteria to be included in this meta-analysis. The studies had been published between 2014 and 2020. The sample size of the articles varied from 737 to 408,931 individuals, and the age of participants ranged from 37 to 69 years. The duration of the follow up of the studies varied from 5 to 22 years. Some studies reported multiple effect sizes in their stratified analysis; for such studies, all suitable data were extracted. The outcome was colorectal cancer in all studies except for the study by Moore et al. [23], which assessed specifically colon cancer. Data for the risk of colorectal cancer for individuals with MUHNW, MHO, and MUHO, compared with subjects with MHNW, were reported in 7 studies with 9 data sets [13, 20–25] and 6 studies with 8 data sets [13, 20, 21, 23–25]. The definition of metabolically unhealthy phenotype was according to the presence of metabolic syndrome [13, 20–22], and having elevated blood glucose (> 125 mg/dL) [23], two studies on females [13, 22], two studies assessed the gender-specific association between obesity phenotypes and the risk of CRC [21, 24], and the rest of the studies reported results for a combination of both genders. Three of the studies were from the USA [13, 22, 23], two were from the UK [20, 25] and two were from Korea [21, 24]. The results of all analyzed articles were controlled for the most potential covariates. Following the NOS scale, all publications indicated good quality (Table 1). Detailed characteristics of the included studies are presented in Table 2.

**Table 1:**
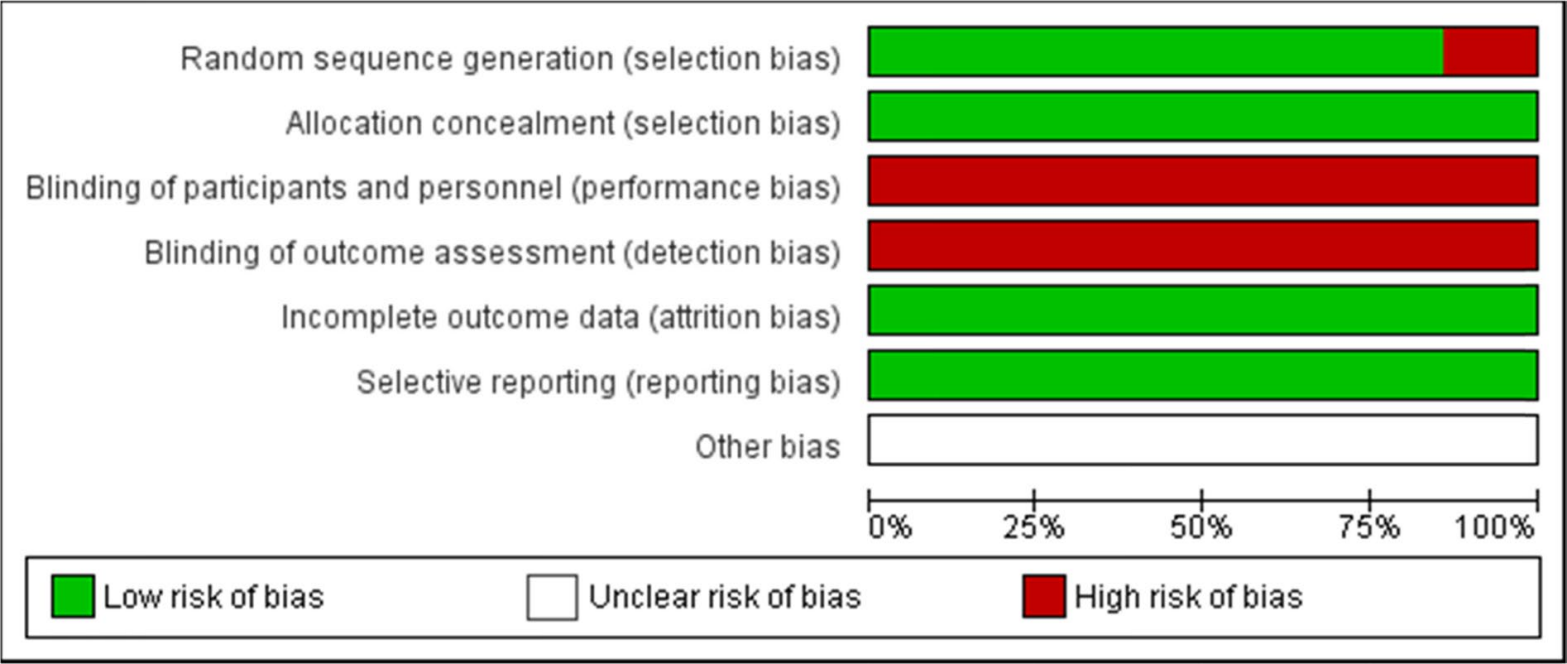
Risk of bias graph: review authors’ judgments about each risk of bias item presented as percentages across all included studies

**Table 2:** Characteristics of studies included in the meta-analysis

Study	Year	Country	Study design	Outcome	Mean or range of age	Sex	Incident cases and sample size (N_CASES_/N_TOTLL_)	Metabolically unhealthy criteria	Definition of BMI categories (kg/m^2^)	Duration of follow up	Adjusted variables in analyses
Moore et al. [23]	2014	USA	Cohort	Colon cancer	55 to 69	Both	Total (147/3763) MHNW (36\1356) MUHNW (3\172) MHO (94\1911) MUHO (14\324)	Metabolically unhealthy phenotype was defined as having elevated blood glucose (> 125 mg/dL)	Normal weight: BMI < 25 kg/m2 Obese: BMI ≥25 kg/m2	1948 to 1970	Adjusted for age, sex, height, education level, alcohol, cigarettes/day, and physical activity plus waist circumference.
Liang et al. [22]	2017	USA	Cohort	Colorectal cancer	66.7 ± 6.9	Female	Total (114/5068) MHNW (64\3358) MUHNW (50\1710)	Presence of metabolic syndrome Based on the criteria of the Adult Treatment Panel III	Normal weight: BMI 18.5 to < 25 kg/m2	1993 to 2015	adjusted for age, ethnicity, smoking, alcohol consumption, physical activity, total energy intake, dietary fiber, percent calories from fat, family history of colorectal cancer, NSAIDs use, and treatment
Murphy et al. [25]	2016	UK	Cohort	Colorectal cancer	57.6 ± 6.4	Both	Total (1474) MHNW (101/232) MUHNW (158/291) MHO (93/214) MUHO (385/737)	Based on the distribution of C-peptide concentration amongst the control population, and participants were classified as metabolically healthy if below the first tertile of C-peptide and metabolically unhealthy if above the first tertile.	Normal weight: BMI < 25 kg/m2 Obese: BMI ≥25 kg/m2	1992 and 2000	was conditioned on matching factors, with additional adjustment for height, smoking status, physicalactivity,educationlevel,alcoholconsumption,anddietaryintakesoftotalenergy,redandprocessedmeats,andfibre
Shin et al. [24]	2017	Korea	Cohort	Colorectal cancer	NR	Male	Total (2951/183,921) MHNW (830\ 94,885) MUHNW (1000\38,218) MHO (362\ 21,828) MUHO (759\28,990)	Those with none of the three metabolic disease components were considered metabolically healthy	Normal weight: BMI < 25 kg/m2 Obese: BMI ≥25 kg/m2	9 years	Adjusted for age and sex, smoking, drinking, exercise, and income.
						Female	Total (2009/225,010) MHNW (700\100,341) MUHNW (597\50,404) MHO (228\34,601) MUHO (484\39,664)				
Kabat et al. [13]	2018	USA	Cohort	Colorectal cancer	62.3 ± 7.2	Female	Total (311/13,535) MHNW (111\4612) MUHNW (20\563) MHO (70\3419) MUHO (110\4941)	Presence of metabolic syndrome Based on the criteria of the Adult Treatment Panel III	Normal weight: BMI 18.5 to 25 kg/m2 Obese: BMI ≥30 kg/m2	1993 and 1998	Adjusted for age, smoking status, alcohol intake, physical activity, aspirin intake, dietary calcium intake, dietary folate intake, caloric intake, oral contraceptives, hormone therapy, family history of colorectal cancer in first-degree relative, education, ethnicity
Cao et al. [20]	2020	UK	Cohort	Colorectal cancer	56.3 ± 8.1	Both	Total (NR/223,030) MHNW (NR/90510) MUHNW (NR/36347) MHO (NR/26094) MUHO (NR/70079)	Presence of metabolic syndrome Based on the criteria of the Adult Treatment Panel III	Normal weight: BMI 18.5 to 25 kg/m2 Obese: BMI ≥30 kg/m2	2006 t0 2016	Adjusted for sex, age, ethnicity, Townsend deprivation index, qualification, employment status, alcohol intaking, smoking status, hormone therapy use, oral contraceptive use and menopause after excluded females with history of hysterectomy.
Cho et al. [21]	2020	Korea	Cohort	Colorectal cancer	57.5 ± 8.0	Male	Total (4136/166,925) MHNW (935/44,194) MUHNW (1625/60,416) MHO (314\13,824) MUHO (1262\48,536)	Presence of metabolic syndrome Based on the criteria of the Adult Treatment Panel III	Normal weight: BMI 18.5 to 25 kg/m2 Obese: BMI ≥30 kg/m2	2009 to 2015	Adjusted for baseline age, income, smoking, alcohol drinking, and presence of inflammatory bowel disease
						Female	Total (2727/152,427) MHNW (708\49,383) MUHNW (1019\50,609) MHO (253\14,733) MUHO (747\37,702)				

*MHNW* metabolically healthy normal weight, *MUHNW* metabolically unhealthy normal weight, *MHO* metabolically healthy obesity, *MUHO* metabolically unhealthy obesity, *BMI* body mass index, *NSAIDs* non-steroidal anti-inflammatory drugs, *CRC* colorectal cancer

### Findings from the meta-analysis

**Fig. 2:**
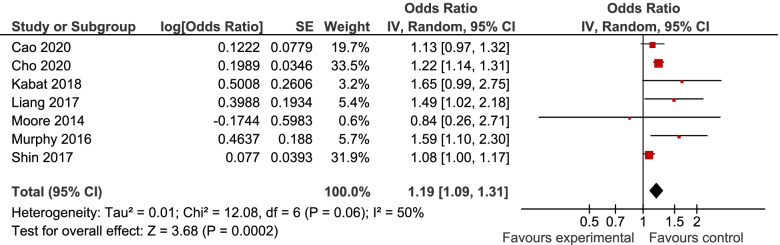
Meta-analysis for the association of MUHNW phenotype, compared with individuals with MHNW, with odds of CRC stratified by study design

**Fig. 3:**
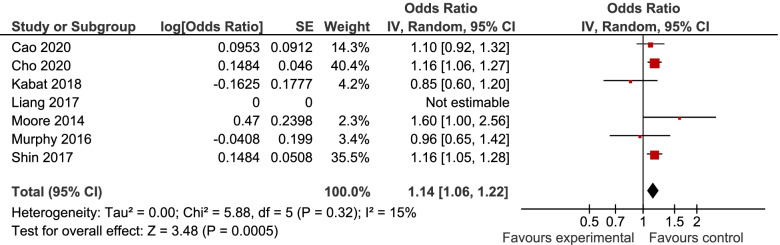
Meta-analysis for the association of MHO phenotype, compared with individuals with MHNW, with odds of CRC stratified by study design

**Fig. 4:**
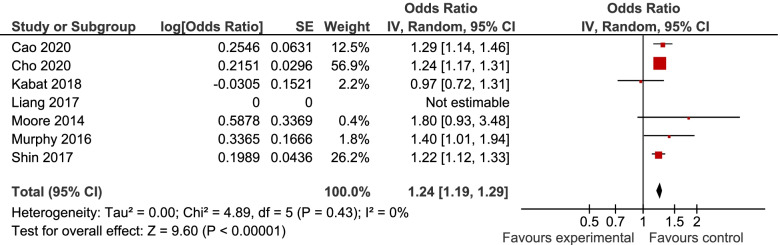
Meta-analysis for the association of MUHO phenotype, compared with individuals with MHNW, with odds of CRC stratified by study design

**Fig. 5:**
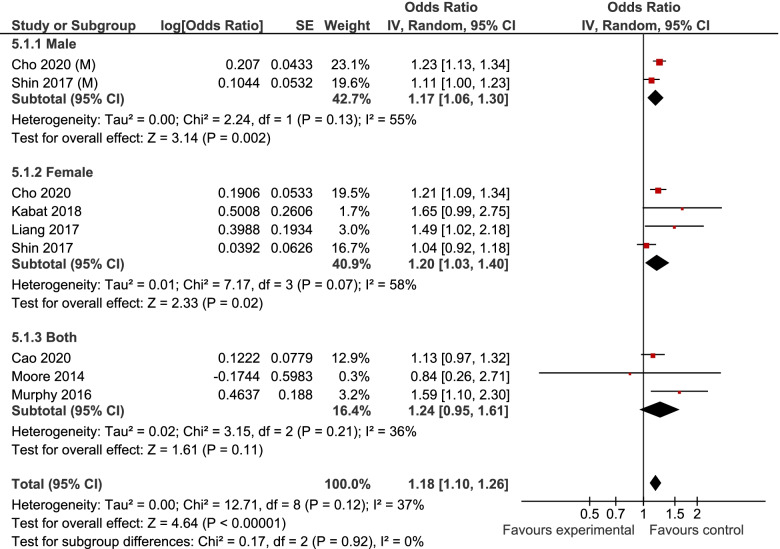
Meta-analysis for the association of MUHNW phenotype, compared with individuals with MHNW, with odds of CRC stratified by gender

**Fig. 6:**
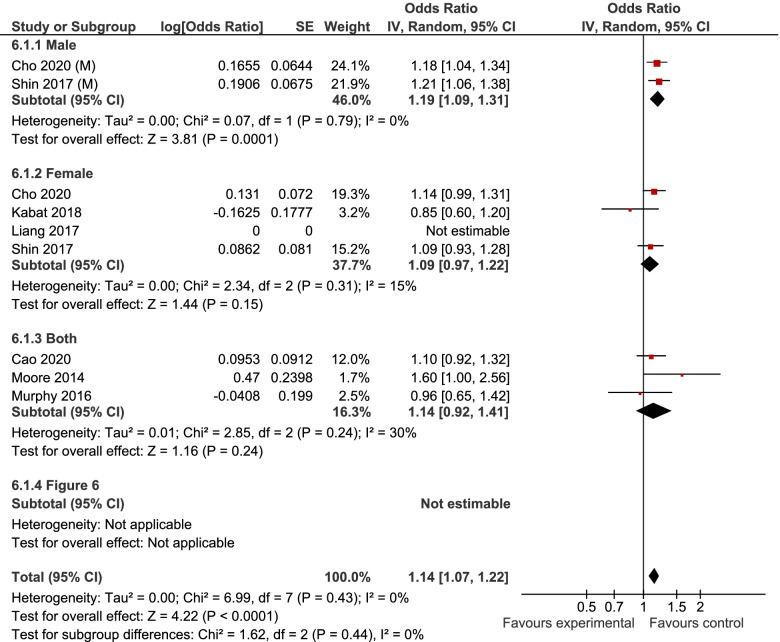
Meta-analysis for the association of MHO phenotype, compared with individuals with MHNW, with odds of CRC stratified by gender

**Fig. 7:**
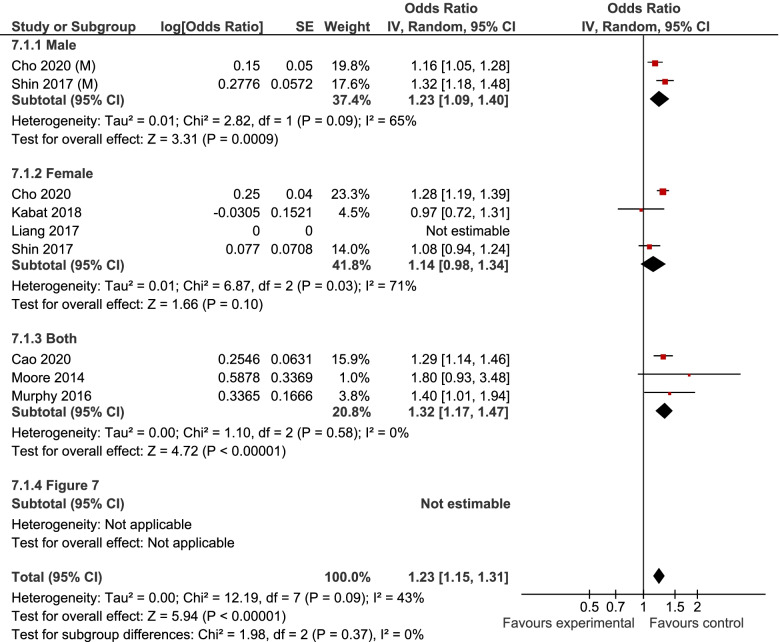
Meta-analysis for the association of MUHO phenotype, compared with individuals with MHNW, with odds of CRC stratified by gender

7, 6, and 6 studies were included in the analyses of MUHNW, MHO, and MUHO, respectively. Compared with individuals with MHNW, those with MUHNW (OR = 1.19, 95% CI = 1.09–1.31; Fig. 2) [13, 20–25], MHO (OR = 1.14, 95%CI = 1.06–1.22; Fig. 3), or MUHO (OR = 1.24, 95%CI = 1.19–1.29; Fig. 4) phenotypes were significantly at an increased risk of CRC. Low heterogeneity was observed in the analysis of MUHO (I^2^ = 0%), whereas moderate heterogeneity was evident in other analyses (I^2^ = 50%). MUHNW (Fig. 5), MHO (Fig. 6), or MUHO (Fig. 7) was associated with an increased risk of CRC in males. By comparison, MUHNW (Fig. 5), but not MHO (Fig. 6) or MUHO (Fig. 7), was associated with a higher risk of CRC in females. Findings showed that the association between metabolic phenotypes with the risk of CRC did not depend on a single study. The pooled effect size ranged from 1.18 (95% CI 1.08–1.27) to 1.32 (95% CI 1.13–1.51) for MUHNW analysis (Supplemental Fig. 1), ranged from 1.11 (95% CI 1.02–1.20) to 1.15 (95% CI 1.10–1.15) for MHO analysis (Supplemental Fig. 2) and ranged from 1.21 (95% CI 1.15–1.28) to 1.23 (95% CI 1.19–1.28) for MUHO analysis, showing the reliability of the results (Supplemental Fig. 3).

### Publication bias

**Fig. 8:**
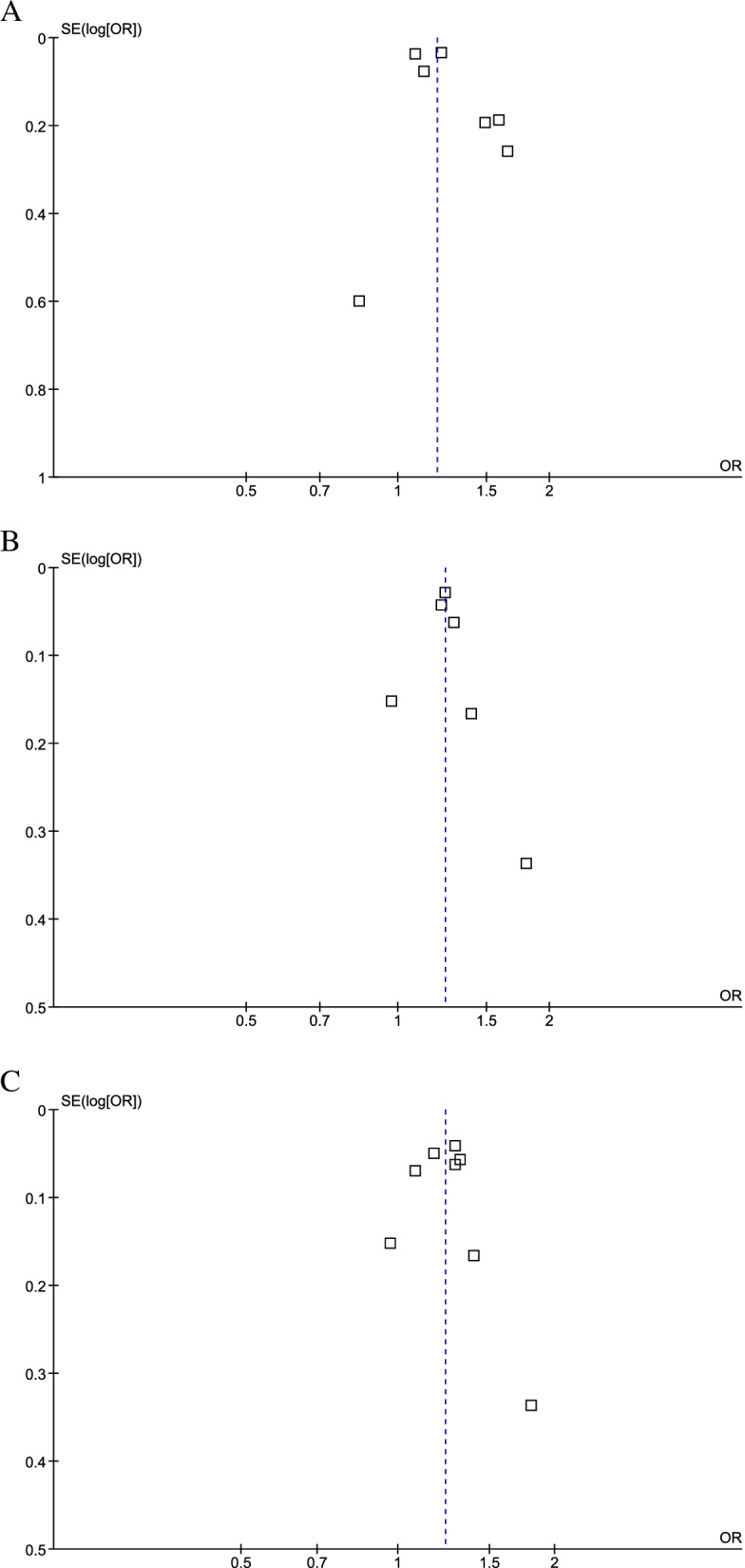
Funnel plot for publication bias in studies investigating the relation of MUHNW (**A**), MHO (**B**), and MUHO (**C**) to the risk of colorectal cancer

No evidence for publication bias was detected based on Egger’s regression test for all analyzes (Fig. 8).

## Discussion

Findings showed being metabolically unhealthy can put people at greater risk for CRC despite having normal weight. We also demonstrated that MHO is not a benign condition as individuals with obesity were at greater risk for colorectal cancer regardless of healthy/unhealthy metabolic conditions. After subgroup analysis by gender, associations remained significant for males. However, the elevated risk of CRC associated with MHO and MUHO was not significant in female participants. This review also highlighted limitations and knowledge gaps of the existing literature.

Previous meta-analyses have also refuted that obesity is a benign condition in absence of metabolic disturbance, suggesting both obesity and poor metabolic health can affect the development of chronic conditions including hypertension [26] and chronic kidney disease [12]. In line with this, we additionally observed the adverse effect of obesity on the development of CRC may be partially offset by metabolic health. By contrast, some studies are indicating an unhealthy metabolic profile completely outweighs the impact of obesity on the risks and progression of certain diseases. The greater risk of cardiovascular disease [27], liver, stomach, prostate, and bladder cancers [20] have been only observed in MUHO but not MHO.

The exact mechanism linking obesity and poor metabolic health to CRC remains unclear, although several possibilities and potential pathways have been proposed. Obesity is positively associated with increased *APC* mutations, reported as gatekeepers in the early stages of the colorectal adenoma-carcinoma sequence [28, 29]. However, poor metabolic health is associated with insulin resistance and consequently activation of insulin-like growth factor-I and epidermal growth factor receptor (EGFR) [30, 31]. EGFR is involved in *K*-*ras* mutation, an essential component for the development and progression of CRC to the advanced stages [32]. Considering these points, it is probable that obesity triggers the early stages of adenoma initiation and development to CRC, while metabolic abnormalities may be responsible in both the early and late stages of CRC progression, but maybe more involved in the late stages. The lower risk of CRC in MHO individuals compared to MUHO can be justified by previous studies which have indicated although MHO individuals accumulate high body fat, they display a better insulin sensitivity, lower inflammatory biomarkers, and high adiponectin levels [33]. Genetic susceptibility, histological characteristics, and geographical locations may partly determine the metabolic health in MHO individuals [34]. Some studies have focused on how dietary intakes could affect CRC initiation and progression by considering different obesity phenotypes. A previous study has shown that adherence to Mediterranean diet or Dietary Approaches to Stop Hypertension (DASH) style diet - which are in accordance with “healthy” pattern and can justify the positive effect in reducing the risk of CRC [35] - was not associated with MHO phenotypes in men > 45 years and premenopausal women [36]. Another study reported that higher pro-inflammatory diet was associated with higher odds of unhealthy phenotype in overweight/obese individuals [37] and a meta-analysis of 40 studies indicated that inflammatory diets such as western-style and alcohol-consumption patterns were associated with an increased risk of CRC, whereas, the healthy dietary pattern was associated with a decreased risk of CRC [37] These results suggest that there is no relationship between metabolically healthy/unhealthy obese individuals and healthy dietary intakes, so they are at a greater risk for CRC.

Concerning subgroup analysis, the greater risk for CRC observed in males compared with females can be justified through several genetic and epigenetic factors [38]. For instance, one explanation may be related to the hormonal status as estrogen and its receptors have shown protective effects in the initiation and progression of CRC [39]. In support of this hypothesis, the results of the Women’s Health Initiative study demonstrated that hormonal replacement therapy can mitigate the risk of colon and rectal cancer by, respectively, 30 and 43% [40]. Apart from estrogen, both insulin and insulin-like growth factor axis may also act differently by sex in CRC carcinogenesis [41].

Obesity was measured based on BMI which is not a very valid indicator of body composition compared to the dual-energy X-ray absorptiometry (DEXA) as a gold standard. However, if the participants with MHO or MUHO had higher lean mass than that of normal-weight participants, the observed associations for the risk of CRC would have been attenuated toward the null. Moreover, the cut-points used to define obesity were different in the included studies. However, this was done to capture ethnicity differences as some ethnic groups have shown a higher risk of weight-related diseases at lower BMI values. Additionally, studies used different guidelines to distinguish metabolic healthy and unhealthy individuals such as ATP III, and so on. Even in studies that used ATP III, some of them only relied on one or a few criteria of metabolic syndrome to determine metabolically abnormal individuals, whereas others considered all the six criteria provided by ATP III. Therefore, it might be difficult to make direct comparisons among these studies. Although reported findings were all conditioned on certain confounders, covariates have widely differed across studies. Measurements were also done at a baseline time point, which cannot capture body weight and metabolic change throughout the study. In previous studies, about 30 to 50% of MHO transitioned to a metabolically unhealthy state, whereas 25 to 30% of MUHO recovered their metabolic health [42–46]. However, the majority of included studies did not reflect the longitudinal change in participants’ body weight and laboratory findings during follow-up. As data regarding trajectories of either BMI or metabolic health were not available, included studies could not properly distinguish between contributing/confounding roles of metabolic status. Another limitation is that we restricted the systematic review and meta-analysis to the use of cohort studies, which are prone to recall and selection bias. The current review is strengthened by applying the most robust approach of meta-analysis for evidence synthesizing and using an established questionnaire of NOS to critically appraise the quality of the evidence. The other strength is the large pooled sample size that can ensure statistical power of findings.

## Conclusions

Individuals with metabolic abnormality, although at a normal weight, have an increased risk for CRC. Moreover, obesity is associated with CRC irrespective of metabolic status. Since the relationship between metabolic phenotypes of obesity and cancer risk has not been extensively investigated by systematic reviews and meta-analyses, the current study offers novel insights into the joint effect of obesity and metabolic abnormality on colorectal cancer risk, which could potentially inform public health practice to keep metabolic healthy, even with normal weight. To uncover the etiological characteristics of metabolic phenotypes an important step forward may be to include different and alternative definitions/criteria of metabolic status for comparison purposes.

## Supplementary Information

**Additional file 1: Supplemental Figure 1**: Sensitivity analysis in studies investigating the association of MUHNW phenotype, compared with individuals with MHNW, with odds of CRC.

**Additional file 2: Supplemental Figure 2**: Sensitivity analysis in studies investigating the association of MHO phenotype, compared with individuals with MHNW, with odds of CRC.

**Additional file 3: Supplemental Figure 3**: Sensitivity analysis in studies investigating the association of MUHO phenotype, compared with individuals with MHNW, with odds of CRC.

**Additional file 4: Supplemental Table 1**. The risk of bias of included studies.

**Additional file 5: Supplemental Table 2**. Risk of bias summary: review authors’ judgements about each risk of bias item for each included study.

## Data Availability

All data generated or analysed during this study are included in this published article [and its supplementary information files].

## Abbreviations

CRC: Colorectal cancer
MUHNW: Metabolically unhealthy normal-weight
MHO: Metabolically healthy obesity
MUHO: Metabolically unhealthy obesity
MetS: Metabolic syndrome
PRISMA: Preferred Reporting Items for Systematic Reviews and Meta-Analyses
RR: Relative risk
HR: Hazard ratio
OR: Odds ratio
CIs: Confidence intervals
NOS: Newcastle-Ottawa Scale
REM: Random-effects model
EGFR: Epidermal growth factor receptor
DEXA: Dual-energy X-ray absorptiometry
