# Supplementary figures and images for: Metabolic phenotypes and risk of colorectal cancer: a systematic review and meta-analysis of cohort studies
Source: BMC Cancer. 2022 Jan 21;22:89. doi: 10.1186/s12885-021-09149-w (PMC8781040; doi:10.1186/s12885-021-09149-w)

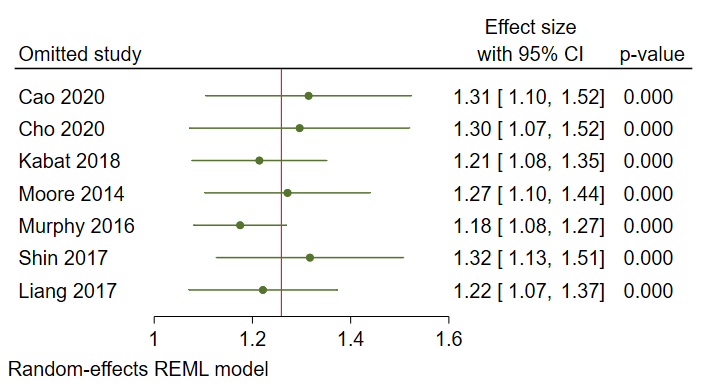

Supplement: Supplementary file 1 — Additional file 1: Supplemental Figure 1: Sensitivity analysis in studies investigating the association of MUHNW phenotype, compared with individuals with MHNW, with odds of CRC. [file 12885_2021_9149_MOESM1_ESM.png]

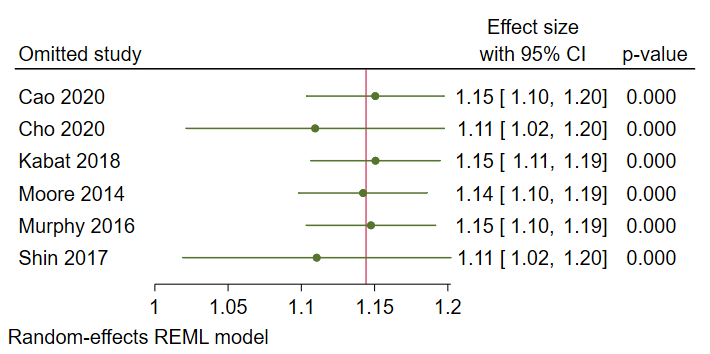

Supplement: Supplementary file 2 — Additional file 2: Supplemental Figure 2: Sensitivity analysis in studies investigating the association of MHO phenotype, compared with individuals with MHNW, with odds of CRC. [file 12885_2021_9149_MOESM2_ESM.png]

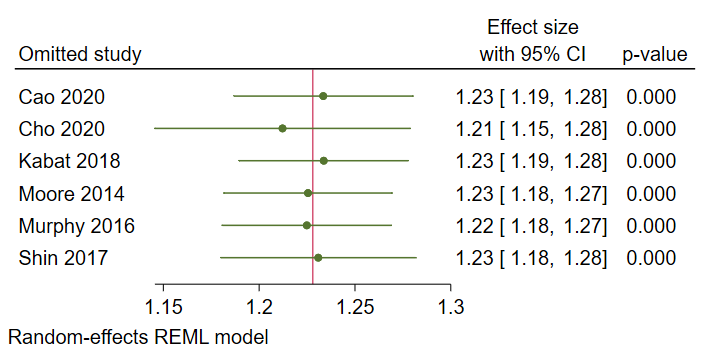

Supplement: Supplementary file 3 — Additional file 3: Supplemental Figure 3: Sensitivity analysis in studies investigating the association of MUHO phenotype, compared with individuals with MHNW, with odds of CRC. [file 12885_2021_9149_MOESM3_ESM.png]

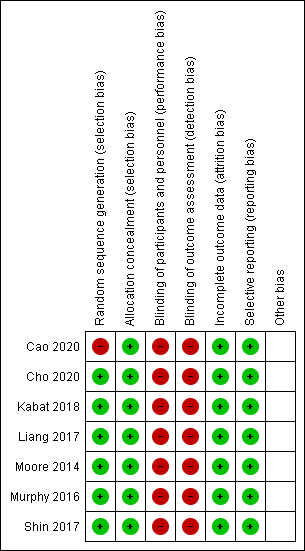

Supplement: Supplementary file 5 — Additional file 5: Supplemental Table 2. Risk of bias summary: review authors’ judgements about each risk of bias item for each included study. [file 12885_2021_9149_MOESM5_ESM.docx]
