# Supplementary material for: Metabolic phenotypes and risk of colorectal cancer: a systematic review and meta-analysis of cohort studies
Source: BMC Cancer. 2022 Jan 21;22:89. doi: 10.1186/s12885-021-09149-w (PMC8781040; doi:10.1186/s12885-021-09149-w)
Supplement: Supplementary file 4 — Additional file 4: Supplemental Table 1. The risk of bias of included studies. [file 12885_2021_9149_MOESM4_ESM.docx]

| Author, Year (Study name) | Random Sequence generation | Allocation  concealment | Blinding of participants and personnel | Blinding of outcome assessment | Incomplete outcome data | Selective  reporting | Other sources of bias |
| --- | --- | --- | --- | --- | --- | --- | --- |
| Moore et al. | Low | Low | High | High | Low | Low | Unclear |
| Liang et al. | Low | Low | High | High | Low | Low | Unclear |
| Murphy et al. | Low | Low | High | High | Low | Low | Unclear |
| Shin et al. | Low | Low | High | High | Low | Low | Unclear |
| Kabat et al. | Low | Low | High | High | Low | Low | Unclear |
| Cao et al. | High | Low | High | High | Low | Low | Unclear |
| Cho et al. | Low | Low | High | High | Low | Low | Unclear |
